# Genetic changes in the genus *Vitis* and the domestication of vine

**DOI:** 10.3389/fpls.2022.1019311

**Published:** 2023-02-28

**Authors:** Ruth Elena Guzmán-Ardiles, Camila Pegoraro, Luciano Carlos da Maia, Antônio Costa de Oliveira

**Affiliations:** Plant Genomics and Breeding Center, Federal University of Pelotas, Pelotas, RS, Brazil

**Keywords:** grape, quality, key genes, evolution, origin

## Abstract

The genus *Vitis* belongs to the Vitaceae family and is divided into two subgenera: *Muscadinia* and *Vitis*, the main difference between these subgenera being the number of chromosomes. There are many hypotheses about the origin of the genus, which have been formed with archaeological studies and lately with molecular analyses. Even though there is no consensus on the place of origin, these studies have shown that grapes have been used by man since ancient times, starting later on its domestication. Most studies point to the Near East and Greece as the beginning of domestication, current research suggests it took place in parallel in different sites, but in all cases *Vitis vinifera* (L.) subsp. *sylvestris* [*Vitis vinifera* (L.) subsp. *sylvestris* (Gmelin) Hagi] seems to be the species chosen by our ancestors to give rise to the now known *Vitis vinifera* (L.) subsp. *vinifera* [=sativa (Hegi)= caucasica (Vavilov)]. Its evolution and expansion into other territories followed the formation of new empires and their expansion, and this is where the historical importance of this crop lies. In this process, plants with hermaphrodite flowers were preferentially selected, with firmer, sweeter, larger fruits of different colors, thus favoring the selection of genes associated with these traits, also resulting in a change in seed morphology. Currently, genetic improvement programs have made use of wild species for the introgression of disease resistance genes and tolerance to diverse soil and climate environments. In addition, the mapping of genes of interest, both linked to agronomic and fruit quality traits, has allowed the use of molecular markers for assisted selection. Information on the domestication process and genetic resources help to understand the gene pool available for the development of cultivars that respond to producer and consumer requirements.

## 1 Introduction

A group of fruit-bearing plants of the genus *Vitis*, of the Vitaceae family is called vine (Wen, 2007), considered one of the oldest crops in the history of mankind, because there is evidence of the use of the fruits since man is considered a collector (Moore, 1991; Chen and Manchester, 2007). Initially it was cultivated in places with hot summers and cold and humid winters, but with the generation of new cultivars it has been possible to find it in the most diverse environments (Alleweldt and Possingham, 1988). Vine is currently cultivated in all continents of the world reaching a total area of 6,950,930 hectares and 78,034,332 tons harvested in 2020, thus constituting one of the most economically important crops (FAO - Food and Agriculture Organization of the United Nations, 2022).

Although *Vitis vinifera* is the best known, 100 species belonging to the genus (The Plant List, 2013) have been identified so far, most of them found in the wild (Bornice et al., 2013). After the devastation of vines in France, hybridization of *V. vinifera* cultivars with wild American Vitis species, in order to get phylloxera resistant and good fruit quality genotypes, were conducted with no success (Walker et al., s.d.). Later on, the use of American species as rootstock began to be a common practice (Pongracz, 1983; Reynolds, 2015). Also, as traits of agronomic interest were recognized, the formation of hybrids between American and *V. vinifera* species were seen as an alternative for the introgression of target genes (Pommer et al., 2003). Although the environment where Asian species are found is different from other producing areas, they constitute a genetic resource for obtaining cultivars resistant to different diseases that affect the crop (Riaz et al., 2018). The mapping of the grape genome has helped to find molecular markers for several traits of interest (Emanuelli et al., 2010; Röckel et al., 2020), important for marker-assisted selection or understanding the phylogenetic relationships between species within the genus (di Gaspero et al., 2000; Aradhya et al., 2008; Myles et al., 2011; Bornice et al., 2013).

Due to the great historical, social and economic importance of this world crop, investigations in the most diverse areas have been carried out trying to elucidate aspects such as origin, domestication, genetic resources, among others (NCBI). All those efforts have contributed to the formation of scientific resources to obtain improved cultivars. However, the abundant collection of existing information is dispersed, making it difficult or time-consuming to consult them. In this sense, the present review appears as a tool to assist both in the development of future research projects and for decision-making in breeding programs.

## 2 Origin and evolution

The genus *Vitis* belongs to the Vitaceae family and comprises more than 60 interfertile species (This et al., 2006). With a few exceptions, most authors divide this genus into two subgenera: *Muscadinia* and *Vitis* (or *EuVitis*), with three and 108 recognized species respectively, with *Muscadinia* species having 2n= 2x= 40 chromosomes and *Vitis*, 2n= 2x= 38 chromosomes (Olien, 1990). Nevertheless, a few consider *Muscadinia* to be a different genus (Olmo, 1995). Wild species of this genus are distributed mainly, but not exclusively, in the northern hemisphere of the earth’s globe with different climatic conditions (Winkler et al., 1974; Hardie and Obrien, 1988; Soejima and Wen, 2006; This et al., 2006). For this reason, this hemisphere is, from the beginning, considered as the place of origin of the genus *Vitis*, but there are differences when specifying the continent in which this happened, there being two or three assumptions in this regard: (1) Asia, (2) America and (3) Eurasia. Thus, after examining the intraspecific variation of 30 species of the subgenus *Vitis*, in agreement with Wei et al. (cited by Wan et al., 2008), Péros et al. (2011) suggested Asia as a place of origin from where it dispersed into Europe and North America during the Pleistocene, but inconsistencies in cpDNA and nuclear DNA data were found. The American lineages studied were older than the Asian ones, but the results were found inconclusive due to previous thought of the genus being of Asian origin (Zecca et al., 2012). On the other hand, pollen remains show that in Georgia (Eurasia) the warm climatic conditions of the early Paleolithic period (32480 - 30180 BC) allowed the appearance of wild grape species (Bar-Yosef et al., 2011), while the genus *Vitis* had its origin in Central America during the late Eocene (Liu et al., 2016), thus agreeing with Zhukovsky (1965).

The studies defining the place of origin lacked sufficient accessions both for the genus *Vitis* and neighboring genera (Liu et al., 2016). The study of 111 accessions indicated that the origin of the genus is North America. and that its diversification into the two subgenera took place in the late Eocene, to later spread to Europe and later to Asia *via* North Atlantic Land Bridges (NALB) or long-distance intercontinental dispersion (LDD). However, a consensus on the origin of the genus has not yet been reached, and some authors suggest the existence of simultaneous centers of origin on the mentioned continents (Wen et al., 2018). What seems certain is that the *Muscadinia* subgenus is endemic to the Southeast of the United States of America (Olien, 1990; Schuck et al., 2014). Even without a mutual agreement on the origin of the genus *Vitis*, all studies have been of great importance in the construction of its phylogeny in order to know the wild and cultivated species and the relationship between them, such as the conservation of the germplasm of plants cultivated worldwide.

In a genomic study, was found that 11.9% of the SNPs predated the speciation of the *Vitis* genus and differentiation from the *Muscadinia* grapes (Magris et al., 2021)

### 2.1 Domestication

Even though the beginning of the domestication of vine species is not completely elucidated, archaeological and archaeobotanical evidence gives an idea of the chronology of the same and the places where it took place. In Mediterranean Anatolia, seeds and fruits of *V. vinifera* and *V. sylvestris* dating from ca. 18200 - 11800 BC, belonging to the Paleolithic period, were found both in the carbonized and mineralized states (Martinoli, 2004), showing that since then the fruits were already consumed. With this, it is possible that the inhabitants of that time also discovered fermentation, but since the stone containers and the juice were consumed immediately (McGovern et al., 1997), the probability of finding containers with organic remains that confirm this hypothesis, is very low (McGovern, 2003).

The first archaeological evidence of fruit uses in a processed form dates back to the early Neolithic period (7000 BC) in China, in the province of Henan, where it was used in combination with rice, honey and other fruits in the elaboration of fermented beverages (McGovern et al., 2004). However, in the years 6000 – 5800 BC, in the South Caucasus region of Georgia, the beginning of viniculture seems to have taken place, as there are indications of fermented grape drinks (McGovern et al., 1996; McGovern et al., 2017). This practice was later extended to northern Iran, where chemical analyzes revealed wine making and storage in the years 5400 – 5000 BC, in the village of Hajji Firuz Tepe (McGovern et al., 1996; Peiró et al., 2018). On the other hand, there is an indication that at the end of the fifth millennium BC, in Dikili Tash, in northern Greece (Wagner, 1967), the use of grape juice was not only for winemaking purposes (Valamoti et al., 2007). Also, in Armenia, there are traces of wine making in 4000 BC (Barnard et al., 2011).

On the other hand, in the same places where viniculture appeared, both seeds and/or pollen of *Vitis vinifera* sp. *sylvestris* as from *Vitis vinifera* sp. *vinifera* dating from the Middle and Late Neolithic period, affirming the great importance of the species *Vitis vinifera* sp. *sylvestris* among all wild Eurasian species to obtain *Vitis vinifera* sp. *vinifera* as a domesticated species (McGovern et al., 2017). From the late Neolithic period (approximately 4000 - 3000 BC) wild grape seeds were found in Greece and Western Europe, and pollen from domesticated grapes in Greece (Hansen and Renfrew, 1978; Marinval apud This et al., 2006; Bar-Yosef et al., 2011). Meanwhile, in Georgia and Turkey, grape seeds domesticated in 6000 BC were reported by Marinval (This et al., 2006), evidencing the impact of man on this plant species. Thus, the use of fresh and processed grapes is not in itself evidence of domestication and extensive cultivation of the vine (McGovern, 1995; Valamoti et al., 2007), but the presence of Archaeobotanical remains, in addition to archaeological remains, indicate that initially the wine may have been made from wild grapes and later, in parallel with domestication.

Thus, several authors agree that the domestication of the vine began in the Near East and Greece (Zohari, 1986; Núñez and Walker, 1989; Olmo, 1995; McGovern, 2003; Myles et al., 2011), from where in the years 3500 - 3000 BC, the practice was gradually spread across the south. From the west of the Fertile Crescent (Jordan Valley, Egypt, lower Mesopotamia) into South Asia, the domesticated species was introduced to Kashmir, between 1700 and 1000 BC, and shortly thereafter to East Asia in 1200 BC in Japan and in the 4th century BC in China (Royer, 1988; Lone et al., 1993; This et al., 2006; Jiang et al., 2009). Subsequently, it spread around the Mediterranean following the main civilizations (Olmo, 1995; Myles et al., 2011; McGovern, 2019), reaching Western Europe, in the year 800 BC (McGovern, 2003). The spread of grape cultivation, led to viniculture, so in the south of France the oldest finds date from 500 to 400 BC, while reports reveal that in the years 138 and 119 BC, viniculture was thriving in Uzbekistan (Jiang et al., 2009). In this way, Greece was able to import wine from other wine regions (Foxhall, 1998).

The domestication of the species allowed breeding by selection, giving rise to the first cultivars, whose names were given by the Roman Empire (27 BC), which in turn expanded its cultivation throughout its jurisdiction, reaching most places in Europe where it is currently grown (Roxas apud. Pagnoux et al., 2015). After the fall of the western empire, during the Middle Ages (476 - 1453 AD) grape cultivation was spread in Northern Europe by the Catholic Church through the Crusades and the spread of the Christian faith, and to North Africa, Spain and the Middle East by Muslims during Islamic expansion (Royer, 1988; This et al., 2006). During this period, trade was common, and there may also be an exchange of genetic material between the Near East and the Aegean world, thus cultivated grape seeds dating from the 7th century (601 - 700 AD) found in the Greek sanctuary at Samos, mark the historical situation of the place and where the pilgrims came from (Pagnoux et al., 2015).

The arrival of the culture in America happened at the end of the 15th century with the discovery of the new world by the Spaniards, being introduced by English, French and Dutch missionaries through seeds and cuttings of *Vitis vinifera* (Dix and Magness, 1937; This et al., 2006). However, several factors prevented the extensive cultivation of the species, such as the poor soils of California (Gerrath et al., 2015), the local pests and diseases, the different climates to those which they were adapted (Dix and Magness, 1937; Gerrath et al., 2015). After 200 years of failed attempts, American horticulturists decided to domesticate wild American species and in the year 1830, American varieties already had a name and were propagated, later (approximately 1850) hybrid varieties of American species and *Vitis vinifera* appeared (Dix and Magness, 1937).

Years later, in the 19th century, colonists moved North American species (such as *Vitis riparia* and *Vitis labrusca*) to Europe (Cornu, 1880; Pommer et al., 2003) and in 1863, in a greenhouse in England, *Phylloxera* sp. was first observed in European viticulture (Cornu, 1880). In 1866 unknown causes caused the death of vine fields in the lower part of the River Rhône, in France, the damage was replicated in different wine-growing areas, and in 1879 a loss of half a million hectares of vine was reported (Cornu, 1880). This devastation came to an end in the year 1900, having as a solution the use of American species as a rootstock for French varieties (Gale, 2002; Cousins, 2005). The loss of European viticulture was not only in economic terms, but above all in a reduction in the genetic diversity of cultivated and wild species, resulting in a rearrangement of the gene pool (Arnold et al., 2005; This et al., 2006; Terral et al., 2010). In addition to phylloxera, other diseases such as powdery mildew and downy mildew entered Europe with the introduction of the American species (Arnold et al., 2005; This et al., 2006). In the same century, European varieties were introduced in South Africa, Australia and New Zealand (Royer, 1988; This et al., 2006). Subsequently, the American species, like *V. labrusca* and *V. rotundifolia*, were used for the development of hybrid cultivars in order to obtain resistance to certain pests and diseases and the traits of the fruit of *Vitis vinifera* (Pommer et al., 2003; Schuck et al., 2014).

#### 2.1.1 Sugar content

The sugar level and firmness of the berry were characters chosen by our ancestors not only for the sweetness in direct consumption and the durability in the transport of the fruits, but also for the traits that this type of fruit gives to fermented beverages (Zhang et al., 2006; Chen et al., 2018). For this reason, cultivated species of *Vitis vinifera* have a higher concentration of soluble sugars in the fruit than wild species (Xin et al., 2013), in addition to a higher content of structural carbohydrates, which give the berry firmness.

With this selection, several genes that participate in carbohydrate metabolism have been favored: hexokinase (*HT4*), 6-phosphofructokinase (*PPFTK4, PPFTK6*), polyol/monosaccharide transporters (*PMT3*), sucrose phosphate synthase (*SPS1*), sucrose synthase (*SUSy1*) and hexose transporters (*HT8; HT15*) (Xin et al., 2013). Of this last group of genes, *SWEET1* was indicated as the most important candidate in the domestication process (Zhou et al., 2017). This gene encodes the bidirectional transport of sugar, specifically glucose, independent of pH (Chen et al., 2010; Chen et al., 2012). Several authors saw its expression increase as it approached veraison (Zhou et al., 2017), specifically on the seed (Rupnik-Cigoj et al., 2018) or on young and old leaves (Chong et al., 2014). Thus, the specific expression of the gene may be dependent on the variety, or species, in this sense, based on the haploid structures, *SWEET1* was suggested as a differential in the phenotype between table grapes and wine grapes (Zhou et al., 2017). On the other hand, *VvSWEET1, VvSWEET2a, VvSWEET2b, VvSWEET4, VvSWEET7, VvSWEET10, VvSWEET11, VvSWEET15, VvSWEET17, VvSWEET17a, VvSWEET17d, Vv01s0146g00260, Vv17s0000g00820* and *Vv17s0000g00830* have been described by researchers as important in the sugar transport in fruits during its development (Chong et al., 2014; Lecourieux et al., 2014; Zhang Z et al., 2019) and for having a role in pest and disease resistance (Chen et al., 2010; Chong et al., 2014), but it was not specified as an object of evolution in the culture of grapes (*Vitis vinifera*).

#### 2.1.2 Flower sex

The vine flower is described as being hermaphroditic, but it was not always like that, in fact, this characteristic is considered one of the most important traits achieved with the domestication process (Zhou et al., 2017) and, therefore, one of the most studied. In this sense, there are different hypotheses on the genetic basis of the sex determination of vine flowers. Sex is determined by a single locus with three alleles (placed in order of dominance): male (M), hermaphrodite (H), and female (F) (Levadoux, 1946; Antcliff, 1980). This was stated by different authors, after mapping with molecular markers (Dalbó et al., 2000; Riaz et al., 2006; Marguerit et al., 2009; Fechter et al., 2012), which located the sex expression locus or sex determining region (SDR) in the binding group 2 or chromosome 2, according to the IGGP nomenclature, between the markers *VVIB23* (Marguerit et al., 2009; Fechter et al., 2012; Picq et al., 2014) and *VVMD34* (Lowe and Walker, 2006; Riaz et al., 2006). On the other hand, an epistatic effect from another locus has been proposed (Carbonneau, 1983), but this hypothesis has not been confirmed by other researchers.

The ABCD model for flower development was proposed for the vine, agreeing that between the establishment and specification of the flower type, the ABCDE genes intervene in the development of floral organs (Ramos et al., 2014; Coito et al., 2018). Dioecy in *Vitis sylvestris* has been identified as the product of a failed development of female organs in the case of male flowers (Caporali et al., 2003), or of male organs in female flowers (Gallardo et al., 2009). Thus, many authors identified male and female sterile candidate genes in the SDR, like *VviYABBY3* (YABBY transcription factor-coding gene), *VviAPT3* (adenine phosphoribosyltransferase) for female-sterility and *VviINP1* (inperturate pollen1) INDEL and WRKY for male sterility (Badouin et al., 2020; Massonnet et al., 2020; Zou et al., 2021). Moreover, a transcription factor of the PLATZ gene family, VviPLATZ1, also localized to the Vitis SDR, was described as a key regulator of female flower formation, and the loss of its function results in the development of reflex stamens, so reducing self-fertilization (Iocco-Corena et al., 2021)

In the first stages of floral differentiation, all flowers are morphologically the same (Ramos et al., 2014). In the process of differentiation and floral development, there are several influencing factors, of which the concentration of hormones plays a main role, as these are the main transducers of genetic information. Thus, the result of development depends on the hormonal balance in the tissues in formation (Srinivasan and Mullins, 1980; Williams, 2000; Chandler, 2011). Therefore, different genes encoding enzymes in cytokinin, ethylene, auxin and gibberellic acid pathways have been considered in studies of genetic determination of the sex of vine flowers. Such as the gene encoding the enzyme 1-aminocyclopropane-1-carboxylic acid synthase, close to SDR (Marguerit et al., 2009), ethylene overproducer-1 (*ETO1*) inside the SDR, 1-aminocyclopropane-1-carboxylic acid synthase (*ACS*) and small auxin up RNAs (SAUR) outside the SDR were pointed as putative candidate genes for the control of sexual traits in grapevine (Carrasco et al., 2020). In this way might be interesting to study whether there is a relationship between this hormones and the genes described earlier, as suggested by Iocco-Coreana et al. (2021).

#### 2.1.3 Fruit color

It is believed that the fruit of the wild subspecies of *Vitis vinifera* had a dark colored pericarp and a non-pigmented mesocarp (Olmo cited by This et al., 2007). This color is the product of a large accumulation of phenolic compounds, which in turn give the fruit antioxidant capacity (Abe et al., 2007). In this sense, the loss of coloration as a consequence of the domestication of the species has been attributed to mutations in the gene that codifies for the production of phenolic compounds.

Still in 1967, Durquety and Destendau (cited by Doligez et al., 2002) indicated the determination of dark color, and therefore of the production of anthocyanin compounds (This et al., 2007), by a single dominant gene, however in grapes pink, there seemed to be the joint action of three dominant genes (cited in Doligez et al., 2002). It was a gene homologous to *VlmybA1-1* (*anthocyanin biosynthesis regulator in Vitis labrusca*), *VvmybA1* with 2 alleles, one of which (allele a) being a product of the *Gret1* retrotransposon insertion at the 5’ end, close to the promoter region of the *UFGT* gene, thus blocking the expression of the *VvmybA1* gene, resulting in the white color of the fruit (Kobayashi et al., 2004; Kobayashi et al., 2005; Kerekes et al., 2019). This loss in anthocyanin production as a result of a mutation was previously suggested by Slinkard and Singleton (1984) who observed that white grapes did not produce other phenols or flavonols in place of anthocyanins. However, only white grapes were observed in individuals homozygous for the a allele, so the white color of the fruit was defined as a recessive trait (Kobayashi et al., 2004; This et al., 2007).

Three macrohaplotypes or haplogroups (N, Rs and B) according to changes in a locus with the *VvMybA* gene family (*VvMybA1*, *VvMybA2* and *VvMybA2*) located on chromosome 2. Types A1 and A2 are functional (their modification directly influences the color of the fruit) and type A3 associated with color determination (Fournier-Level et al., 2010). Also, the diversity in fruit color may be due to mutations at other points in the anthocyanin biosynthesis pathway or in the coding and non-coding regions of the *VvMybA1* gene and by chimeras in the grape skin layers (This et al., 2007; Migliaro et al., 2014; Migliaro et al., 2017; Péros et al., 2015)

The *Gret-1* retrotransposon was not observed in other species of the genus *Vitis*, which indicates that the insertion took place after the separation of *Vitis vinifera* from the North American and Asian species (Mitani et al., 2009), from where crosses between individuals with this mutation and wild ones allowed the amplification in the color of the fruit (Cadle-Davidson and Owens, 2008).

#### 2.1.4 Berry size

As berry size is the product of different physiological processes within the plant and berry, different genes are involved (Muñoz-Espinoza et al., 2016). Thus, during the domestication of the species, alleles of genes involved in each of these processes were selected, which makes the study of this trait very complex. As a product of domestication, Negrul (1946) distinguished three offspring: occidentalis, small berry cultivars from western Europe; orientalis, table grape cultivars with large berries from central Asia and Pontica, intermediate phenotype grown around the Black Sea and eastern Europe.

A QTL coding for seedless grapes, berry size and weight, ripening date, was identified in LG 18, which may indicate the existence of a pleiotropic effect of these characters (Cabezas et al., 2006; Mejía et al., 2007; Costantini et al., 2008). Additionally, the existence of gene regions (LG5, LG11, LG13 and LG15) (Cabezas et al., 2006) and a QTL on chromosome 17 (Doligez et al., 2013) that code for berry size regardless of the presence of seeds were suggested. Candidate markers such as SNPs and InDels were suggested for berry weight, a related trait for berry size, in seedless table grapes on chromosomes 3, 6, 9 and 14, as well as being co-localized with QTLs previously identified for the same trait on LG 8, LG17 and LG 15 (Muñoz-Espinoza et al., 2020).

The determination of fruit size depends mainly on cell division and cell expansion during flowering and the first stages of fruit formation (Coombe, 1992; Muñoz-Espinoza et al., 2016). During ovary development, *VviANT1* (AINTEGUMENTA-like) gene, located on LG 18, showed a possible role in the cell division regulation (Chialva et al., 2016). The gene *VvNAC26* (=*VvNAP*) seems to regulate cell elongation and fruit size during ovule development by regulating the expression of hormone-related genes (Zhang et al., 2021). However, a polymorphism of this gene was identified regarding the size of the berry and the minihaplotype MH5 demonstrated a role in the growth of the fruit (Tello et al., 2015). The expression of this gene differs between seeded and seedless genotypes (Zhang et al., 2021) and a variant of this gene was correlated with small final berry size, thus a reduced gene expression will result in larger berry size (Fernandez et al., 2006). Besides, an interaction of *VvNAC26* with *VvMADS9* (=*VvPI*, PISTILLATA) was observed (Zhang et al., 2021). In addition, transcription factors (bHLH60, bHLH93 and bHLH96) were observed participating during the first stages of fruit formation (Muñoz-Espinoza et al., 2016), one of which, identified as *VvCEB1*, is involved in cell size regulation during berry development in Cabernet Sauvignon and therefore can be used as a marker for this trait (Nicolas et al., 2013). The second phase of berry growth was gained with the process of domestication and occurs at veraison, thanks to the greatly increased expression of two LRR–receptor kinase genes (*VIT_217s0000g05570* and *VIT_217s0000g05580*) localized in chromosome 17 (Magris et al., 2021). Finally, Guo et al. (2019) identified four genes related to berry weight: *VIT_218s0001g01370* (wall-associated receptor kinase 2-like), *VIT_219s0015g00730* (cellulose synthase-like protein e6-like) and *VIT_217s0119g00330* (uncharacterized protein) on chromosomes 18, 19 and 17 respectively and Li et al. (2021) found that *VvSAUR041*, located in the cytoplasm on LG 4, can promote cell expansion, therefore a candidate gene for berry size in grapes. With a transcription analysis, genes associated to transcription regulation, cell wall modification, transport of metal ions, water and organic acids, response to biotic/abiotic stress, protein degradation and protein-kinase activation were identified in seedless grapes (Muñoz-Espinoza et al., 2016).

#### 2.1.5 Seed morphology

Even though this trait is not a target of selection, differences between seeds of wild and cultivated species were found, some authors suggest that it is due to a pleiotropic effect of some gene encoding another trait of interest (Mangafa and Kotsakis, 1996; Terral et al., 2010). Others suggest that the increase in seed size is due to hormonal mechanisms from management and selection processes (Bouby et al., 2013). In any case, although the reasons for this change have not been clarified, given that there are no differences between the places studied, it is believed that there is no direct influence of the environment on the morphology of the seed (Terral et al., 2010).

Several archaeobotanists have described wild grape seeds as round or cordiform, flat on the ventral part, with acute angles, a strongly developed chalaza and with a short tip (Stummer, 1911; Mangafa and Kotsakis, 1996; Bouby et al., 2013). With this, different methods of identification of both subspecies have been proposed, taking into account their proportions in the form of indices (Stummer, 1911; Mangafa and Kotsakis, 1996).

#### 2.1.6 Recent trends

##### 2.1.6.1 Seedlesness

The absence of noticeable seeds in table grapes is a required character by consumers, and therefore, sought after by breeding programs for this type of grape (Ledbetter and Ramming, 1989). This absence of seeds occurs naturally in the fruit under two mechanisms: (1) Parthenocarpy, where fertilization of the ovule is not required for fruit development to occur and the lack of seeds is due to the impairment of meiosis; (2) Stenospermocarpy, in which fruit formation occurs after pollination and fertilization, but the embryo and/or endosperm does not continue to develop (Stout, 1936; Pratt, 1971; Ledbetter and Ramming, 1989; Ramming et al., 2000; Royo et al., 2016). The former occurs rarely in grapes, as in genotypes such as Black Corinth (Ledbetter and Ramming, 1989; Ramming et al., 2000), the second mechanism is more common and, because it is heritable, has little influenced by the environment and does not affect berry growth (Stout, 1936; Pratt, 1971; Ramming et al., 2000), it is widely used as a research tool and is commercially preferred (Notsuka et al., 2001).

In the formation of stenospermocarpic fruits, several quantitative components are involved: fresh weight, dry weight and total number of seeds and trace seeds, degree of integument hardness and degree of endosperm development (Striem et al., 1996), which are governed by different gene regions. Thus, Mejía et al. (2007) found several QTLs in linkage groups (LG) 4, 8, 15, 16 and 18, being the most stable the QTLs of LG 18 (Doligez et al., 2002), which by in turn showed a high LOD score with the SEED DEVELOPMENT INHIBITOR locus (SDI; Lahogue et al., 1998) (Cabezas et al., 2006; Mejía et al., 2007; Mejía et al., 2011; Doligez et al., 2013), explaining more than 70% of the phenotypic variation of seeds (Mejía et al., 2007). However, the absence of seeds had previously been suggested by Roytchev (1998) as a recessive trait that can also be controlled by dominant genes repressing seed formation, while Bouquet and Danglot (1996) ensured the action of three independent recessive genes regulated by a dominant inhibitory gene. Additionally, Notsuka et al. (2001) propossed that the inheritance of this trait happens through a complex system of four dominant genes that code for the inhibition of seed formation, which in turn is controlled by a dominant regulatory gene, with the seeded fruit having the homozygous recessive genotype. Thus, SdI appears as the dominant locus in LG18 (Cabezas et al., 2006; Mejía et al., 2007; Costantini et al., 2008; Mejía et al., 2011; Doligez et al., 2013).

Furthermore, *VvAGL11*, a MADS-box gene, was indicated as the main candidate gene responsible for stenospermocarpy, and the variation in its promoter region is what gives the individual the seedless grape phenotype (Mejía et al., 2011). This variation was identified by Royo et al. (2018) as a missense SNV causing the substitution of Arg-197Leu in *VviAGL11*, disrupting the function of multimeric complexes containing VviAGL11 proteins. In addition, during seed formation, several genes encoding MADS-box transcription factors were identified as important, therefore recommended for molecular studies of the control of the “seedless” condition in grapes. (Wang et al., 2015). It is the case of a class E MADS-box gene, *VvMADS39*, which forms a dimer with *VvAGAMOUS* and when *VvINO* is expressed, together promote ovule abortion and inhibit normal ovule development by the restriction of cellular expansion. The expression of *VvMADS39*, in turn, is regulated by upstream BPC TF and the suppression occurs by the enhancement of histone H3 lysine 27 trimethylation in the promoter region of *VvMADS39* (Zhang et al., 2022). The *VvINO* TF belongs to the YABBY gene family and is encoded by a unique gene placed on top of chromosome 1 (Di Rienzo et al., 2021). Others YABBY genes were also identified as important on seedless genotypes, like *VvYABBY4* which seems to restrict endosperm cell expansion during seed growth and *VvYABBY5*, that even though its expression is not directly related to ovule abortion, appears to play a role in seed development (Zhang S et al., 2019). The latest was also found to be related to grape berry shape on table grapes (Wang et al., 2020). Moreover, some B3 domain transcription genes were described as candidate genes in the determination of seedless or seeded berries, thus *RAV3*, *RAV4* and *REM2* were indicated as having a role in seed abortion of seedless grape cultivars, while *ABI3-1*, *ABI3-3*, and *VAL1* may promote normal seed development (Ahmad et al., 2019)

On the other hand, molecular markers linked to the SdI locus have been developed by different researchers, including SCAR, SCC8 (Lahogue et al., 1998) and SCF27 (Mejía and Hinrichsen, 2003), SSR markers VMC16f11 (Arroyo-García and Martínez-Zapater, 2004) and VMC7f2 (Cabezas et al., 2006), STS marker p3_VvAGL11 (Mejía et al., 2011). All of these have been tested for molecular-assisted selection in F1 hybrid progeny from different crosses for early detection of the “seedless” condition, reaching different conclusions on the reliability of marker use depending on the genotypes used (Mejía and Hinrichsen, 2003; Fatahi et al., 2004; Cabezas et al., 2006; Korpás et al., 2009; Mejía et al., 2011; Akkurt et al., 2012). In this sense, several studies have shown differences in the inheritance of this trait depending on the parents and their genetic background (Stout, 1936; Pratt, 1971; Striem et al., 1996; Notsuka et al., 2001; Doligez et al., 2002; Mejía et al., 2007; Wang et al., 2015).

##### 2.1.6.2 Disease resistance

The fungi *Uncinula necator, Botrytis cinerea* and *Plasmopara viticola* are pathogens that attack the flower and fruit of the grape, resulting in economic loss for the producer. In the case of table grapes, the visual quality of the bunch is lost, due to the presence of few healthy berries, in addition to allowing the excessive use of agrochemicals harmful to human health (Moss, 2008). In wine grapes, enzymes and other substances produced by *Botrytis cinerea* in grape berries, the action of bacteria associated with the fungus (Meneguzzo et al., 2006) and the production of secondary metabolites by the plant, such as methyl salicylate under the attack of *Plasmopara viticola* and *Guignardia bidwellii* (Poitou et al., 2021) affects the composition and quality of the wine (Fedrizzi et al., 2011; Steel et al., 2013). However, these differences in aromatic compounds in wines obtained from fruits affected by *Botrytis cinerea* and *Erisiphe necator* proved to be, to a certain degree, positive, when is a noble rot (Lopez Pinar et al., 2016; Dankó et al., 2021).

Thus, obtaining resistant cultivars to these pathogens has been the objective of several breeding programs, though resistance and good organoleptic characteristics is difficult to achieve through plant hybridization. In the case of the vine, several mechanisms of resistance to the fungus have been identified, depending on the plant-pathogen interaction. The first mechanism occurs in the first moments of infection, when the plant synthesizes proteins related to pathogenicity (PR), such as chitinases, b-1,3-glucanases and thaumatin-like proteins (Monteiro et al., 2003) and also, genes that encode these PRs have been transferred to genetically modified cultivars (Kikkert et al., 2000; Harst et al., 2000; Yamamoto et al., 2000). In addition, during the first contact of the pathogen with the plant, the joint activation of flavonols and stilbenes biosynthesis takes place within the plant, and the intensity and duration of these will result in the phenotype of resistant cultivars, allowing the vine to produce phenolic compounds of low molecular weight or stilbenics, commonly called phytoalexins (Ciaffi et al., 2019). Some genes that participate in the biosynthesis of phytoalexins are phenylalanine ammonia-lyase (*pal*), chalcone synthase (*chs*) and stilbene synthase (*stsy*) (Sparvoli et al., 1994), which in addition of being cloned from grape plants, stsy was transferred to tobacco, rice, barley and maize DNA (Bavaresco and Fregoni, 2001). Xu et al. (2019) reported that the overexpression of *VpSTS29/STS2* (stilbene synthase gene) in *Vitis vinifera* increases the concentration of stilbene synthase enzymes in the mesophyll, resulting in high production of resveratrol derivatives in the infection zone.

The next is the gene-for-gene mechanism or protein-protein interaction, where the species and genotype of Vitis recognize specific elicitors produced by a particular pathogen, activating pathogen resistance genes (Di Gaspero and Cipriani, 2002). Most R genes code for proteins with a Nucleotide binding site (NBS), leucine-rich repeat region (LRR), Drosophila toll/mammalian interleukin-1 receptor (TIR) ​​or the leucine-zipper/coiled-coil motif (cc) and other kinase domains (Ellis et al., 2000). In *V. amurensis*, Li et al. (2015) after mildew attack identified the expression of 37 R genes homologous to the NBS-LRR RPS2 gene, in addition to five proteins containing R genes -NBS, three -NBS-LRR and 28 –LRR in V. rupestris. Feechan et al. (2013) found that the Run1 locus in *M. rotundifolia* comprises a family of seven putative NB-LRR-like Toll/interleukin-1 receptor (TIR) ​​R genes, one of them *MrRUN1*, which confers resistance to powdery mildew. For *Erysiphe necator*, Yang et al. (2008) identified R genes on chromosomes 4, 5, 7, 9, 12, 13, 15 and 18, while Di Gaspero et al. (2007) on chromosomes 9, 12, 13, 18 and 19, while Weng et al. (2014) identified 318 putative R genes, of which 132 were upregulated in infected grapes, most of them similar to Mlo (downy mildew locus O). Also were found 26 R CNL genes (CC-NB-LRR type) of which two were RPS2 (GSVIVT01021921001, GSVIVT01037631001), 23 R RLP genes, including one BAK1 (GSVIVT01029816001) and two MRH1 genes (GSVIVT01032772001, GSVIVT01021228001). In the Pinot Noir genome, Malacarne et al. (2012) identified 391 R genes encoding proteins with a functional nucleotide binding site (NBS) domain, of which 291 are grouped into 52 clusters (CL) with 2 to 15 genes at an average distance of 8.3 kb, located in the chromosomes 1, 3, 5, 7, 9, 12, 13, 15, 18 and 19, but did not specify the pathogen to which they conferred resistance.

Several gene loci that confer horizontal resistance to *Uncinula necator* have already been described. Such as *Run* (resistance to *U. necator*): *Run1* (identified in *M. rotundifolia*) on chromosome 12 (Dry et al., 2010), *Run2.1* (*M. rotundifolia* ‘Magnolia’), *Run2.2* (*M. rotundifolia* ‘Trayshed’) on chromosome 18 (Riaz et al., 2011), *Run5* (*V. rotundifolia*) (Blanc et al., 2012); *Ren* (resistance to *Erysiphe necator* - syn. *U. necator*): *Ren1* (Asian *V. vinifera* var. Kishmish vatkana) in GL13 (Kozma et al., 2009), *Ren2* (*V. cinerea*) (Dalbó et al., 2001), *Ren3* (Regent hybrid whose pedigree includes *V. aestivalis, V. berlandieri, V.cinerea. V. labrusca. V. lincecumii, V. riparia and V. rupestris*) (Fischer et al., 2004), *Ren4* (*V. romanetii*) on chromosome 18 (Riaz et al., 2011), *Ren6* (*V. piasezkii*) on chromosome 9 and *Ren7* (*V. piasezkii*) on chromosome 19 (Pap et al., 2016).

Loci with genes that confer vertical resistance to *Plasmopara viticola* were also identified: Rpv (resistance to *P. viticola*): *Rpv1* (*M. rotundifolia*) on chromosome 12 (Abdullaevich Abdullaev et al., 2020), *Rpv2* (*M. rotundifolia* ‘Trayshed’), *Rpv3* (two populations with a background of Vitis americana) on chromosome 18 (Fischer et al., 2004; Bellin et al., 2009). In addition, QTLs that also confer resistance to both powdery mildew and downy mildew have been mapped on chromosomes 4, 7, 9, 12, 13, 15, and 18 (Di Gaspero et al., 2007; Yang et al., 2008). In *V. riparia* QTLs for downy mildew were identified in LG 9 and 12 (Marguerit et al., 2009), 12 and 4 (Moreira et al., 2011) and Regent hybrid in LG 4 and 18 (Fischer et al., 2004; Welter et al., 2007). A new *Erysiphe necator* resistance QTL was found in a caucasian variety of *Vitis vinifera*, at the Ren1.2 locus, appearing as a novel resistance gene candidate to be used in breeding programs (Possamai et al., 2021).

With this knowledge, in recent years several repeated sequences close to the R genes, which can serve as markers, have been identified. Such are the resistance gene analogs (RGAs) in wild and hybrid species (Di Gaspero and Cipriani, 2002), for downy mildew and powdery mildew (Pauquet et al., 2001; Fischer et al., 2004; Welter et al., 2007; Bellin et al., 2009; Marguerit et al., 2009).

Additionally, other molecular markers linked to resistance genes were also identified, among which we found: for the *Run1* gene, the RFLP markers GLP1-12 and MHD145 (Donald et al., 2002), VMC8g9 and VMC4f3.1 (Barker et al., 2005) and SSR markers VMC4f3.1 and VMC8g9 (Schuck et al., 2011), SSR markers UDV020a, VMC9h4.2 and VMCNg4e10.1 (Hoffmann et al., 2008), for *Rpv1*, VMC1g3.2 (Merdinoglu et al., 2003), for *Ren1* SSR markers UDV020a, VMC9h4.2 and VMCNg4e10.1 (Hoffmann et al., 2008). Marker identification allows for marker-assisted selection (SAM), allowing for a faster improvement process. Also, with the identification of these markers, it was possible to test different strategies of introgression of resistance genes in cultivated vine species (Agurto et al., 2017). Katula-Debreceni et al. (2010) were able to detect pyramid resistance to powdery mildew in the BC5 hybrid family, and there are even reports of the use of multiple pyramided resistance genes used as a genetic improvement method (Eibach et al., 2009). On the other hand, the use of fast-generation “microvines” is being widely used for the construction of lines carrying polygenic resistance without compromising the organoleptic characteristics of the fruit (Chaïb et al., 2010).

Finally, with the increase of information, it was possible to build genetic and physical maps, the latter using a bacterial artificial chromosome (BAC) library (Barker et al., 2005; Di Gaspero et al., 2007) which are tools important in the localization of resistance genes or related to it.

## 3 Conclusions

Being among the first domesticated crops, grapes have changed to satisfy human requirements, increasing in fruit size, sugar content, fruit color and fruit morphology. A narrow genetic variability was imposed by soil and air borne diseases imported from the American continent. Although great changes have been obtained so far, the possibilities emerging with the novel biotechnological tools can provide a wider portfolio of grape and wine varieties to humankind.

## Author contributions

RG-A conceived the idea. RG-A, CP contributed to the writing. CP, LM and AO reviewed and edited the manuscript. All authors contributed to the article and approved the submitted version.
